# Response of brain metastasis from lung cancer patients to an oral nutraceutical product containing silibinin

**DOI:** 10.18632/oncotarget.7900

**Published:** 2016-03-03

**Authors:** Joaquim Bosch-Barrera, Elia Sais, Noemí Cañete, Jordi Marruecos, Elisabet Cuyàs, Angel Izquierdo, Rut Porta, Manel Haro, Joan Brunet, Salvador Pedraza, Javier A. Menendez

**Affiliations:** ^1^ Department of Medical Oncology, Catalan Institute of Oncology, Doctor Josep Trueta University Hospital, Girona, Spain; ^2^ Girona Biomedical Research Institute (IDIBGi), Girona, Spain; ^3^ Department of Medical Sciences, Medical School, University of Girona, Girona, Spain; ^4^ Department of Radiology, Diagnostic Imaging Institute, Doctor Josep Trueta University Hospital, Girona, Spain; ^5^ Department of Radiotherapy, Catalan Institute of Oncology, Doctor Josep Trueta University Hospital, Girona, Spain; ^6^ ProCURE (Program Against Cancer Therapeutic Resistance), Metabolism and Cancer Group, Catalan Institute of Oncology, Girona, Spain; ^7^ Department of Pneumology, Doctor Josep Trueta University Hospital, Girona, Spain

**Keywords:** non-small cell lung cancer, brain metastasis, silibinin, STAT3, Legasil

## Abstract

Despite multimodal treatment approaches, the prognosis of brain metastases (BM) from non-small cell lung cancer (NSCLC) remains poor. Untreated patients with BM have a median survival of about 1 month, with almost all patients dying from neurological causes. We herein present the first report describing the response of BM from NSCLC patients to an oral nutraceutical product containing silibinin, a flavonoid extracted from the seeds of the milk thistle. We present evidence of how the use of the silibinin-based nutraceutical Legasil^®^ resulted in significant clinical and radiological improvement of BM from NSCLC patients with poor performance status that progressed after whole brain radiotherapy and chemotherapy. The suppressive effects of silibinin on progressive BM, which involved a marked reduction of the peritumoral brain edema, occurred without affecting the primary lung tumor outgrowth in NSCLC patients. Because BM patients have an impaired survival prognosis and are in need for an immediate tumor control, the combination of brain radiotherapy with silibinin-based nutraceuticals might not only alleviate BM edema but also prove local control and time for either classical chemotherapeutics with immunostimulatory effects or new immunotherapeutic agents such as checkpoint blockers to reveal their full therapeutic potential in NSCLC BM patients. New studies aimed to illuminate the mechanistic aspects underlying the regulatory effects of silibinin on the cellular and molecular pathobiology of BM might expedite the entry of new formulations of silibinin into clinical testing for progressive BM from lung cancer patients.

## INTRODUCTION

Brain metastases (BM) represent an unmet need in current oncologic care of cancer patients, particularly in non-small cell lung cancer (NSCLC) patients [1, 2]. Approximately 10% of NSCLC patients will have BM at presentation and 25–40% will develop BM during the course of the disease [3, 4]. Lung cancers are the primary source of BM and account for 40–55% of cases, followed by breast cancer (15–20%), and melanoma (5–10%) [5].

The prognosis of NSCLC patients with BM is poor, with a median overall survival time of 7 months [6]. Only limited treatment options exist upon the occurrence of BM and the use of chemotherapy is challenging due to the blood-brain barrier, which restricts the delivery of therapeutic drug concentrations inside the central nervous system [1]. There is a strong need for the identification of novel treatment modalities to improve the high morbidity and mortality of NSCLC BM patients.

Silibinin (or silybin) is a natural polyphenolic flavonoid isolated from seed extracts of the herb milk thistle (*Silybum marianum*). Preclinical studies show that silibinin has a strong efficacy to target migratory and invasive characteristics of cancer cells, demonstrating anticancer effects *in vitro* and *in vivo* [7, 8]. We present two cases of NSCLC where supplementation with a silibinin-based nutraceutical showed promising activity against BM in patients that progressed after standard treatment regimens and presented reduced performance status.

Because BM patients have an impaired survival prognosis and are in need for an immediate tumor control, our current findings and further mechanistic studies into the regulatory effects of silibinin on the cellular and molecular pathobiology of BM promise to yield exciting biological breakthroughs and valuable clinical insights in the ideal management of BM from lung and other cancers.

## RESULTS

### Silibinin supplementation shows activity against progressive brain metastases of NSCLC patients

**Figure 1:**
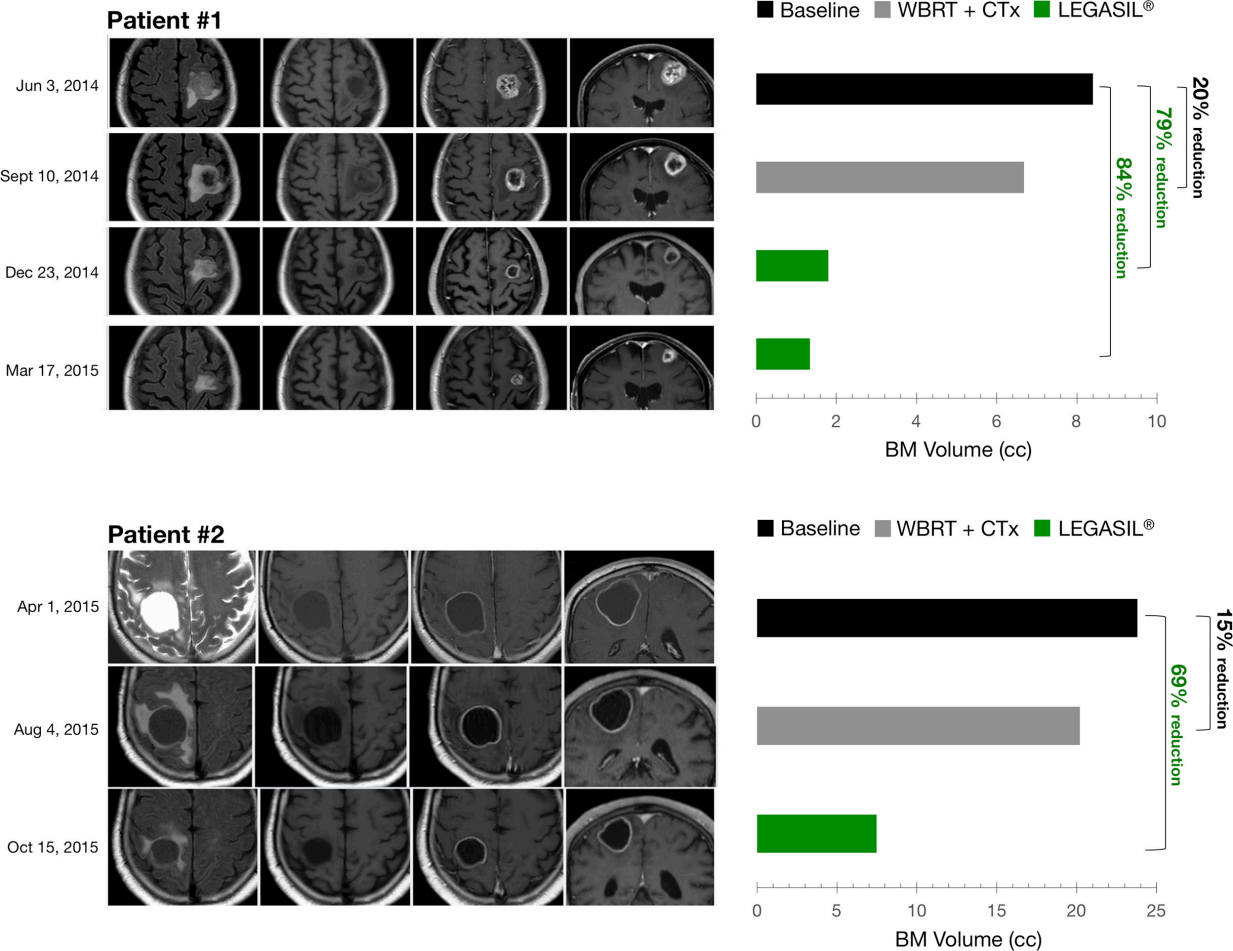
*Left panels*. MR Imaging of brain metastasis changes following CTx+WBRT and Legasil^®^ treatments Four sequences are shown prior to and post-treatments (from left to right): axial FLAIR, axial T1-weighted, post-contrast axial T1, and post-contrast coronal T1. *Right panels*. Volumetric responses of brain metastasis following CTx+WBRT and Legasil^®^ treatments. CTx: chemotherapy, WBRT: whole brain radiotherapy treatment.

A 62-year-old Caucasian female never-smoker presented with an episode of myoclonic seizure of the upper right extremity and decreased level of consciousness in May 2014. A magnetic resonance imaging (MRI) of the brain in June 2014 revealed five brain metastases (the largest measuring 24 × 25 × 28 mm) (Figure 1, *top*). Her work-up included a computed tomography (CT) scan, which showed multiple bilateral lung nodules of different sizes, solid and with well-defined margins, and some with a tendency to coalesce, all suggestive of malignancy. The largest lesion was located in the right lower lobe, measuring 27 mm. Multiple bilateral mediastinal lymph nodes were also noted; additionally, a left adrenal nodule was diagnosed. Lung biopsy was consistent with lung adenocarcinoma. No epidermal growth factor receptor (EGFR) activating mutations or echinoderm microtubule-associated protein like 4-anaplastic lymphoma kinase (EML4-ALK) translocations were identified.

After initial neurological improvement with dexamethasone, the patient underwent whole brain radiotherapy treatment (WBRT) with 30 Gy in 10 fractions. Chemotherapy treatment was started one week later, consisting of the alkylating agent carboplatin (AUC 5) and the folate antimetabolite pemetrexed (500 mg/m^2^) every 3 weeks. On the sixteenth day post third cycle, the patient consulted for worsening of neurological symptoms. A brain MRI in September 2014 revealed persistence of the lesions with a minor decrease of the volume and an increase of the surrounding edema (Figure 1, *top*). A body CT scan showed stability of the multiple neoplastic lung nodules. Neurological deterioration persisted despite high doses of dexamethasone, and the patient presented an Eastern Cooperative Oncology Group (ECOG) score of 3. Oncologic treatments were stopped at this point and the patient was transferred to a palliative care unit to continue receiving best supportive care.

The patient asked about additional treatment options to improve her symptoms. A compassionate use of the experimental nutraceutical product containing silibinin (Legasil^®^) was offered and informed consent was obtained according to article 37 of the 2013 Declaration of Helsinki [9]. One-week dosing titration (see Patients and methods section for details) was performed to achieve the desired total dose with no undesirable side effects. The patient was discharged from the palliative care unit two weeks later due to clinical improvement. Four weeks after initiating silibinin supplementation, the patient consulted with her oncologist. After discussing treatment options with the patient, an adjusted pemetrexed monotherapy (400 mg/m^2^) was started in combination with Legasil^®^ (5 capsules/day). After 3 cycles, a brain MRI performed in December 2014 revealed a marked reduction in lesion volume and a decrease in brain edema (Figure 1, *top*). The patient received an additional cycle of pemetrexed and continued with Legasil^®^ alone. A repeat brain MRI in March 2015 revealed a mild reduction in the size of the left frontal lesion and in the extent of the surrounding edema, and a CT scan showed stable pulmonary disease. Lung progression was evident in June 2015 (increase of 20% of the target lesions) and a brain MRI revealed the persistence of partial response in 4 of 5 BM (only one brain lesion increased from 10 × 9 mm to 14 × 15 mm, but without neurological symptoms). The patient is currently receiving a second line treatment with ECOG 0.

A 67-year-old Caucasian male heavy current smoker (>90 pack-years consumption) presented with left hemiparesis and walking instability in March 2015. A brain MRI performed in April 2015 showed an isolated parietal cystic brain lesion with peripheral enhancement (Figure 1, *bottom*). The lesion presented a moderate surrounding edema and a moderate mass effect over the right lateral ventricle. A CT scan revealed a multilobulated nodular lesion located in the right upper lobe measuring 31 mm, and a spiculated nodular lesion in the right middle lobe (20 mm). Ipsilateral hilar and mediastinal lymphadenopathy were also noted. A bronchoscopic biopsy confirmed a diagnosis of lung adenocarcinoma. Analysis for EGFR mutations and EML4-ALK translocation was not possible due to insufficient tumor material for molecular testing.

The patient underwent WBRT with 30 Gy in 10 fractions. The patient had an ECOG score of 2 and pemetrexed (500 mg/m^2^) monotherapy was initiated, requiring dose adjustment (400 mg/m^2^) in the second cycle because of hematologic toxicity (febrile neutropenia grade 4). After three cycles, a CT scan showed disease progression with an enlargement of the right upper lobe lesion (39 mm) and the appearance of multiple bilateral lung nodules. A brain MRI showed a subtle reduction of the volume of the cystic brain metastasis but an increase of the surrounding brain edema. The lesion showed persistence of the peripheral enhancement and persistence of the mass effect over the lateral ventricle. Because of a decline in performance status (ECOG 3) and no response to first line treatment, best support care was offered to the patient. The patient asked about additional treatment options.

The patient started Legasil^®^ titration but he stopped at 3 capsules/day (1-1-1) because of diarrhea grade 2 with 4 capsules/day dosage. After 4 weeks of treatment, the patient showed clinical improvement of neurological symptoms. After 8 weeks of Legasil^®^ monotherapy, a brain MRI revealed a considerable decrease of the volume of the lesion and in the extent of the edema, with a clear reduction of the mass effect over the ventricle (Figure 1, *bottom*). There was a persistence of the peripheral enhancement of the lesion. A CT scan revealed stable pulmonary disease (increase of 16% of the lung lesions). Over this time, his performance status improved to ECOG 1 and treatment options were discussed. The patient is currently receiving a second-line treatment of chemotherapy. Table 1 summarizes the radiological evolution of tumor lesions in the two patients treated with Legasil^®^.

**Table 1:** Radiological evolution of tumor lesions in two NSCLC patients treated with Legasil^®^

Evolution of intracranial disease							
	Patient #1				Patient #2		
	Jun 3, 2014 [Baseline]	Sep 9, 2014 [QT+RT]	Dec 23, 2014 [Legasil^®^]	Mar 12, 2015 [Legasil^®^]	Mar 31, 2015 [Baseline]	Jul 22, 2015 [QT+RT]	Oct 7, 2015 [Legasil^®^]
**AP diameter**	2.4	2.4	1.5	1.2	4	3.5	2.6
**T diameter**	2.5	2.3	1.5	1.6	3.4	3.4	2.4
**CC diameter**	2.8	2.6	1.6	1.4	3.5	3.4	2.4
**Volume**	8.3	6.7	1.8	1.3	23.8	20.2	7.5
**Edema evolution**	Baseline	Increased	Decreased	Decreased	Baseline	Increased	Decreased
**Evolution of extracranial disease**							
	Jun 3, 2014	Sep 9, 2014	Dec 23, 2014	Mar 12, 2015	Mar 31, 2015	Jul 22, 2015	Oct 7, 2015
**Target lesions**							
Right inferior lobe	2.7	2.7	2.2	2.2	3.1	3.9	4.6
Left inferior lobe	2.1	1.9	1.7	1.8	2.0	1.7	1.9
TOTAL SUM	4.8	4.6	3.9	4.0	5.1	5.6	6.5
**Non target lesions**							
Mediastinal adenopathy	P	P	P	P	P	P	P
Hilar adenopathy	P	P	P	P	P	P	P
Left adrenal nodule	P	P	P	P	P	P	P
New lesions	NA	No	No	No	NA	Yes	No
Overall response	NA	SD (5% reduction)	SD (19% reduction)	SD (17% reduction)	NA	PD	SD (16% increase)

All measures are expressed in cm

P: Present, A: Absent, NA: not applicable, PD: progression disease, SD: stable disease

## DISCUSSION

Untreated patients with BM have a median survival of about 1 month, with almost all patients dying from neurological causes [10]. Dexamethasone provides temporary symptomatic relief of central nervous system symptoms related to increased intracranial pressure and edema secondary to BM [2]. WBRT with doses up to 30 Gy is the standard of care for patients with >3 BM and good performance status (Karnofsky Index ≥ 70%) [11]. Stereotactic radiosurgery can be considered for patients with ≤3 BM and measuring less than 3 cm in maximum diameter [2]. Prospective trials demonstrated the activity of first-line chemotherapy for BM of NSCLC, but with median survival of 5–8 months in most cases [1]. In a recent phase II clinical trial, cisplatin-pemetrexed concurrently with WBRT (30 Gy in 10 fractions) yielded a cerebral response rate of 68.3%, with an overall survival of 12.6 months in NSCLC (adenocarcinoma histology) patients with BM at presentation [12]. Pemetrexed monotherapy has demonstrated moderate efficacy and good safety in chemotherapy-naïve ECOG performance status (PS) 2 patients with EGFR wild-type or unknown advanced non-squamous NSCLC [13]. In most cases, patients with BM can only receive 1 cycle or less of chemotherapy due to early death, rapid progression, clinical impairment, or toxicity, and these rapid deteriorations are especially frequent in patients with ECOG PS 2 [14]. Despite front-line treatment activity, treatment of recurrent/progressive BM is more controversial, especially for patients with neurological symptoms and poor performance status where no further treatment (supportive care) is recommended [15]. Accordingly, the ESMO guidelines for metastatic NSCLC recommend best supportive care for ECOG PS ≥ 3 in the absence of documented activating (sensitizing) EGFR mutations [16].

The use of complementary therapies (CoTs) among cancer patients is frequent. A recent study in six Italian oncology departments reported that 37.9% of patients were using one or more types of CoT, with diets and dietary supplements (27.5%) and herbs (10.8%) the more commonly used [17]. Despite the increase in popularity of CoTs, they are still viewed with skepticism by medical professionals and the majority of oncologists recommend complete avoidance of all supplements [18]. Yet, we should acknowledge that plant-derived leading compounds have been historically used for chemotherapy of cancer. In 2013, our group reported potent activity of silibinin in a NSCLC preclinical model of acquired resistance to the EGFR tyrosine kinase inhibitors gefitinib and erlotinib [19–21]. More recently, we reported the case of a heavily pre-treated breast cancer patient with progressive liver failure caused by extensive liver cancer infiltration, which improved after silibinin supplementation [22]. Additionally, a previous work showed that silibinin significantly alleviated neurological deficit and suppressed brain edema in an induced ischemic stroke model in mice [23]. Because of these data, and the lack of any alternative treatment options because of the poor performance status of our patients, we considered that silibinin supplementation could provide some clinical relief.

Our group has recently reviewed the role of silibinin in cancer, and we concluded that preclinical evidence in diverse cancer types suggest that silibinin might be viewed as a natural inhibitor of signal transducer and activator of transcription 3 (STAT3) [24]. STAT3 is constitutively activated in many different cancer types and plays a pivotal role in tumor growth and driving metastasis, including BMs [25]. The expression of activated STAT3 is higher in human melanoma BM specimens than in primary tumors [26]. Inhibition of STAT3 by WP1066 decreased the incidence of BM and increased survival in a preclinical model of breast cancer BM [27]. Furthermore, STAT3 and miR-21 are cooperative regulators of stemness, migration and tumor initiation in lung-derived BM [28]. Inhibition of miR-21 resulted in similar reductions in self-renewal and migration of BM-initiating cells to those achieved with STAT3 knockdown, and knockdown of STAT3 also reduced expression of known downstream targets of miR-21. We showed that silibinin suppresses EMT-driven acquired resistance to erlotinib by reversing the high miR-21/low miR-200c signature *in vivo* [20]. It is noteworthy that the preferential silibinin's ability to impact mechanisms of growth control at the brain site (i.e., brain metastatic colonization) without inhibiting primary tumor (or extra-cranial metastatic disease) reveals a remarkable organ-type specificity that might reasonably involve reactivation of metastasis suppressor genes [29–31] and/or suppression of genes that enable efficient cellular survival and outgrowth of BM-initiating cancer cells during the progression of BM [32–35]. Moreover, it might appear counterintuitive to explain the significantly clinical and radiological improvement of BM from NSCLC patients in terms of STAT3 inhibition because the suppressive effects of the silibinin-based nutraceutical Legasil^®^ on progressive BM occurred without affecting the primary lung tumor outgrowth in NSCLC patients. However, although the ultimate mechanistic aspects underlying the apparently specific anti-BM effects of silibinin remain largely elusive, it should be acknowledged that the brain-specific potentiated effect of silibinin might merely reflect the attenuation of WBRT-activated mitogenic and pro-survival signaling including STAT3 in cancer as well as endothelial cells. Because radiotherapy has been shown to increase the vascularity and invasiveness of surviving EMT-like radioresistant cancer cells, the brain's specific response to silibinin-induced STAT3 blockade might reflect the inhibition of radiation-induced progression (or pseudoprogression) of intracranial lesions in comparison to non-irradiated, STAT3-independent extracranial ones [36–40]. Moreover, STAT3 inhibition most likely does not exert antineoplastic effects by purely cell-autonomous mechanisms [41] as its blockade is expected to limit the production of pro-inflammatory factors, hence reducing local inflammatory reactions, and stimulate the recruitment of immune effectors into the tumor bed and improve immunosurveillance, especially in the context of ongoing anticancer immune responses [42]. Although the brain has long been considered an “immune-privileged” organ with limited capacity for inflammatory response, it is becoming clear that BM harbors an active inflammatory microenvironment that is capable of inducing prominent anti-tumor immune responses [43]. Because established BM contain considerable inflammatory infiltrates composed of various immune cells [44], the marked reduction of the large peritumoral edema on progressive NSCLC BM might reflect how silibinin-induced inhibition of STAT3 may increase the immunogenicity of BM cancer cells via cell-autonomous pathways and/or may favor the cell-nonautonomous reprogramming of the BM microenvironment (e.g., endothelial cells, tumor-associated macrophages, tumor-infiltrating lymphocytes) toward an immunostimulatory state [45–48].

Despite the promising preclinical activity of silibinin, anticancer activity remains to be shown in human trials [8]. This could be explained by the poor water solubility (<0.04 mg/ml) of its flavonolignan structure and subsequent low bioavailability [19]. High oral doses of 13 g of silybin-phytosome daily, in 3 divided doses, has been shown to be well tolerated in patients with advanced prostate cancer and was the recommended phase II dose [49]. Although high-dose oral silybin-phytosome achieved high blood concentrations transiently, i.e., 1 h after the first silybin-phytosome dose silibinin blood levels reached a mean value of 19.7 microM, low levels of silibinin and no significant anti-tumor activity were seen in prostate cancer tissue [50]. By intravenous administration, doses of 20 mg/kg/day of silibinin monotherapy leads to safe, potent and time-dependent *in vivo* anti-viral effects in difficult-to-treat HIV/HCV-coinfected patients [51, 52]. Because repeated intravenous boluses may be problematic for some patients, the results observed strongly suggest that the oral use of a Eurosil 85^®^-based nutraceutical [53] could be the first silibinin formulation that represents “*exciting seeds of change for the prevention and treatment of cancer*” [8].

There is a strong need for the identification of novel treatment modalities to improve the outcomes of the most frequent primary tumors causing BM, such as melanoma and NSCLC. Here, we present evidence of how the use of the silibinin-based nutraceutical Legasil^®^ resulted in significant clinical and radiological improvement of BM from NSCLC patients that progressed after whole brain radiotherapy and chemotherapy and presented reduced performance status. Several preclinical studies have reported that activation of STAT3 is an important driver of BM but none of the drug-like candidates that target STAT3 in pre-clinical models has yet entered into clinical use [54, 55]. A number of preliminary research findings collected in our laboratory begins to suggest that silibinin can exert also STAT3-independent regulatory effects on key genes involved in the promotion and maintenance of cancer stem cells (CSCs) self-renewal during metastatic dissemination as well as in the immune active inflammatory microenvironment [56, 57], which may eventually produce additive or even synergistic anti-BM effects when combined with other therapeutic strategies. Because BM patients have an impaired survival prognosis and are in need for an immediate tumor control, the combination of brain radiotherapy with silibinin-based nutraceuticals might not only alleviate brain edema but also prove local control and time for either classical chemotherapeutics with immunostimulatory effects or new immunotherapeutic agents such as checkpoint blockers to reveal their full therapeutic potential in NSCLC BM patients. If it is proven that silibinin's anti-BM function is causally linked to its ability to deplete the pool of BM-initiating CSCs and disrupt their inflammatory niche and supporting vasculature, new formulations of silibinin such as Legasil^®^ might rapidly entry into the clinic for use in the ideal management of BM from lung and other cancers.

## PATIENTS AND MATERIALS

This study examined two patients with NSCLC BM to identify their responses to Eurosil 85^®^ (Euromed, Mollet del Vallés, Barcelona, Spain), a new formulation of silibinin launched in Spain in January 2014 under the commercial name of Legasil^®^ (Meda Pharma, Rottapharm-Madaus, Barcelona, Spain). Each Legasil^®^ capsule contains 210 mg of Eurosil 85 (60% of silibinin isoforms) which, according to the product patent data, has an increased release rate (80%) and improved absorbability. The product is available without medical prescription as it is considered a nutritional supplement.

*In vivo* studies using NSCLC xenograft models demonstrated that oral gavage administration of silibinin at 100 mg/kg body weight caused highly significant decreases in tumor volume as compared to NSCLC fed controls. According to the body surface area (BSA) method proposed by Reagan-Shaw et al. [58] for an appropriate conversion of drug doses from animal studies to human studies, the corresponding human equivalent dose (HED) is 8.11 mg/kg silibinin. This equates to a 486.49 mg dose of silibinin for a 60 kg individual.

**Figure 2:**
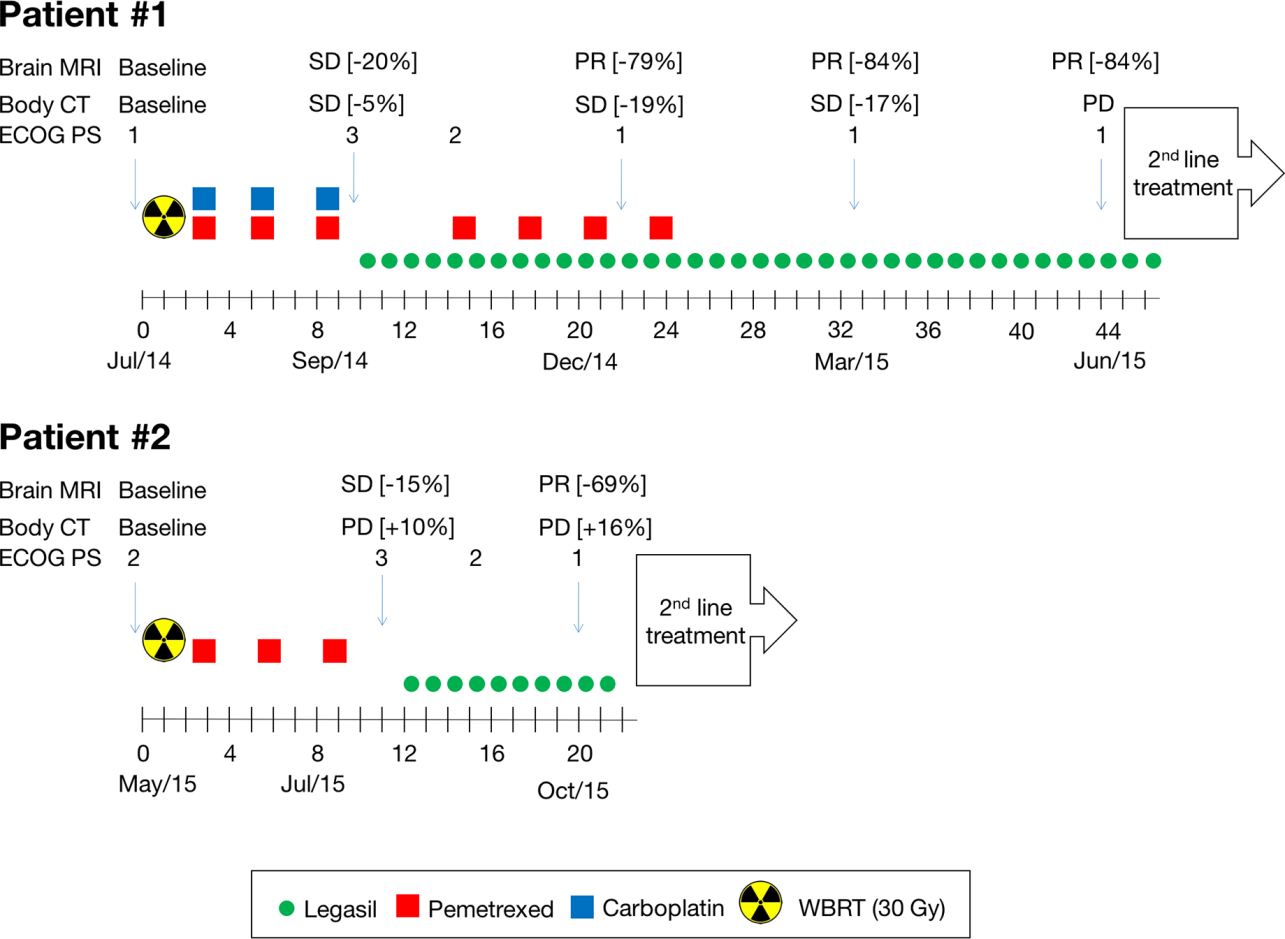
Treatment schema of NSCLC BM patients

Both patients presented clinical impairment despite receiving standard treatment including WBRT and chemotherapy. Both patients accepted this compassionate treatment, and a signed consent form was obtained according to article 37 of the 2013 Declaration of Helsinki before treatment was started [9]. A titration was started with 2 capsules of Legasil^®^ (1-0-1) for 3 days and an additional capsule was then added until a 5 capsules dosage (2-2-1) was achieved or toxicity was observed. At the posology of five capsules a day of Legasil^®^, the nutraceutical provided 1,050 mg of Eurosil 85, which equated to a 630 mg-dose-a day silibinin regimen. The treatment schema for each patient is illustrated in Figure 2.
